# Medical News and Items

**Published:** 1876-06

**Authors:** 


### Medical News and Items.

Do "They Do These Things Better in France"? A writer in the *Soir* remarks that the wretched custom prevails in France of expecting physicians and attorneys to wait for remuneration of professional services for an indefinite period of time. "It often happens that a physician is not paid until a year has elapsed after his visits. What do they expect him to live upon while he is waiting? I recall a remark made by one of my friends on receiving, after three years, the account of his medical attendant: 'I wouldn't have thought it of him!' If the custom of other nations were introduced in France, we should no longer have presented to us the sad spectacle of a man of science, often advanced in years, aroused from sleep in the night and made to ascend and descend several flights of stairs, the payment for whose services is neglected with less scruple than would be entertained for a mere lackey."

THERE IS WAR IN CANADA, between the *Canada Medical Record*, which is supposed to represent the interests of the College of Physicians and Surgeons of Lower Canada, and *L' Union Médicale de Canada*. The subject of contention is a Medical Bill of Reform, presented by Hon. M. Chapleau in the interest of the general profession, who have ventured to operate in the matter without the advice and assistance of the honorable professors of the Medical College, and, in particular, the Editor of the *Record*. The Editor of the *Union Médicale* satirizes his editorial confrère as Jupiter Tonans. "He desires to be esteemed a Jupiter, it is true, but Minerva never sprang, full armed, from his head. The *Medical Record* thinks it monstrous that the property of the College should be transferred to another organization. We should like to know of what this property consists. What is left when you take away its archives, its charter, and its seal? Would you answer by referring to the petty sum which comes largely from the sale of licenses, useless to those who gain them? Distribute this among its members and bury the decrepit organization with an Homeric repast. All's well that ends well. The College is powerless for good or evil. The evil it does is even purely negative, for it cannot prevent the accomplishment of good. Better commit suicide under these circumstances, for then 'life becomes an opprobrium and death a duty.'"

---

A DANGEROUS PRESCRIPTION.—The following prescription was presented at a pharmacy: R, Acid. Chromic., grs. viij; Glycerinæ, ℥j. M. et S. For external use. The clerk dissolved the acid in a vial by the aid of a little water, then added the glycerine and agitated the contents. An explosion occurred which expelled the contents of the vial, but fortunately did not burst the latter, which was left in the hand of the stupefied drug-clerk.—*Pharm. Central.*

THE following books have been donated to the library of the Chicago Medical Press Association.

From Dr. A. Reeves Jackson:

Lewis' Dispensatory.
Turner's Chemistry.
Barton's Lectures on Materia Medica, 2 vols.
Beddoes' Observations.
Carmichael on Venereal Diseases.
Gregory's Practice, 2 vols.
Dewees' Practice, 2 vols.
Package of Journals.

From Dr. R. C. Hamill:

Medical and Surgical History of the Rebellion, 2 vols.
Ninth Census of the United States, Circular No. 4.
Transactions of American Medical Association, 12 vols.
Medico-Chirurgical Review, 1827.
Mackenzie, Diseases of the Eye.
Patent Office Report, 1851 and 1855.

From Dr. J. M. Woodworth, U. S. M. H. S.:

Annual Reports Marine Hospital Service, 1873 and 1874.
Nomenclature of Disease.
Migrants and Sailors.
Hospitals and Hospital Construction.
Contributions to the Study of Yellow Fever.

From State Board of Health of Michigan (Dr. Baker, Sec.):

Michigan State Reports, Nos. 1, 2, 3, 4 and 5.
Health Report for 1872, 1873, and 1874.

EDW. WARREN SAWYER,
*Librarian.*

---

PHOTOGRAPHY AS A MEANS OF DIAGNOSIS.—The photographic process is so acutely sensitive that it will often present vividly certain blemishes which the eye is incapable of detecting. Some years ago a lady had a photograph taken of herself in which the face appeared covered with blotches which could not be seen in the original. The next day they became evident to the naked eye and she finally died of small pox. The photograph had proceeded farther than vision, and recognized in advance, exceedingly delicate light-yellow stains.—VOGEL. *Photography & Chemistry of Light.*

---

THE PHYSICIAN OF THE SULTAN.—The Sultan of Turkey, grateful for the relief which he has had from a recent illness, has just presented his Medical Director General with one thousand Turkish pounds.—*Lyon Médical.*

A Pharmacist with a Catheter in his Hand.—A Pharmacist of Bordeaux sent his son, a student in pharmacy, to catheterize an individual affected with retention of urine. Soon after the patient succumbed. The tribunal has just condemned this student to ten days imprisonment for the injuries inflicted in his ignorance; and a fine of forty-five francs for violation of the law respecting the practice of medicine.—*Lyon Médical.*

---

The City of Paris as a Dissection Centre.—"Even now she is the city which offers the greatest resources for dissection, for nowhere can subjects be found in greater number ; as independently of those which the numerous hospitals furnish, many of the departments around Paris send the bodies of all their dead prisoners to Clamart or École Pratique. The latter is situated in the heart of the Latin Quarter, and, although its appointments are not so complete as the former, still, its proximity to the École de Médecine, and the rooms of a great number of students, causes it to be much frequented. More than a thousand cadavers are yearly required for the uses of this institution, while Clamart demands from two thousand to twenty-five hundred, as, while the former is opened only during the scholastic year, the latter is continually accessible. The charges for dissecting material are now moderate, as both establishments furnish it to the student for the sum of twenty francs ($4.) per term."—*Paris Correspondence Det. Med. Rev.*, April, 1876.

---

The Editor of the *Louisville Medical News*, in referring to the fact that the University of Louisville had decided to abolish the requirement of theses from candidates for graduation, says: "We believe this is the first institution which has taken this step." It cannot be that our friend takes the papers. Rush College, of Chicago, has not required theses for several years.

"Double-Headed Diplomas.—A correspondent from Carthage Ill., writes: 'In your issue of Feb. 26th, you mention "a new, time-saving method of securing a diploma," and credit those Siamese twins institutions at Louisville, Ky., with inaugurating it; but the credit (?) for this innovation belongs farther west. The College of Physicians and Surgeons of Keokuk, Iowa, has had a summer and winter course of lectures in operation for three or four years past, and grants diplomas after an attendance on the two courses, so that a student may commence attending lectures in October and graduate in the following May.' "—*N. Y. Med. Record*, March 11, 1876.

